# Enhancing Human–Robot Compatibility in Shoulder Exoskeletons: Passive Joint Optimization of PPRRRP vs. RRRUP Configurations

**DOI:** 10.3390/biomimetics10120795

**Published:** 2025-11-22

**Authors:** Qiang Cao, Wenhao Shan, Yue Liu, Yongqi Yuan

**Affiliations:** The College of Machine, Shanghai DianJi University, Shanghai 201306, China; caoq@sdju.edu.cn (Q.C.); 15138209350@163.com (W.S.); yq20240901@163.com (Y.Y.)

**Keywords:** shoulder rehabilitation, exoskeleton mechanism, human–robot compatibility, kinematic performance, comparative analysis

## Abstract

This study aims to evaluate the kinematic performance of two shoulder rehabilitation exoskeleton configurations to address the critical challenge of human–robot compatibility. Utilizing Hunt’s mobility formula and task-specific Jacobian analysis, we developed a closed-chain kinematic model integrating transient glenohumeral joint dynamics, validated through force/torque measurements and ANOVA statistical comparisons. The PPRRRP configuration, featuring orthogonally distributed passive prismatic joints, demonstrated superior performance: 40–60% lower interaction forces (${\bar{F}}_{total}=2.66\;N$), near-isotropic manipulability (ellipsoid axis ratio < 1.5), and 60% reduced operational torque (${\bar{T}}_{total}=0.18\;N\cdot m$) compared to RRRUP’s universal joint design. These results establish passive DOF optimization as a viable alternative to actuator-dense systems, diverging from conventional approaches like ARMin-III that prioritize active control. The originality lies in bridging theoretical configuration synthesis with empirical validation, offering a replicable framework for compatibility assessment. This work advances rehabilitation robotics by demonstrating that mechanical transparency—achieved through strategic passive joint allocation—enhances natural movement synergy without compromising stability, proposing hypotheses on energy efficiency and isotropy–fatigue correlations for future exploration. Clinical translation and adaptive impedance control integration are identified as critical next steps to optimize patient-specific rehabilitation outcomes.

## 1. Introduction

The shoulder joint, as the critical enabler of upper limb mobility [1], is frequently impaired by neurological injuries (e.g., stroke) or trauma, severely limiting patients’ functional independence [2]. Conventional rehabilitation relies on clinician-guided manual therapy [3], where therapeutic protocols are designed based on subjective expertise and iteratively refined through outcome assessments [4]. While effective in controlled settings, this approach suffers from practitioner-dependent variability, high labor intensity, and scalability challenges [5], exacerbated by economic constraints and shortages of specialized personnel. Consequently, many patients resort to unsupervised training regimens that lack scientific rigor, often failing to achieve desired therapeutic outcomes [6]. Rehabilitation robotics has emerged as a promising alternative, yet persistent kinematic incompatibility between exoskeletons and the human shoulder complex remains a critical barrier. Initial misalignments during device donning and inherent biomechanical disparities often prevent real-time congruence between the exoskeleton’s pivot and the glenohumeral joint center (CGH), generating parasitic forces/moments at the human–robot interface [7]. These forces compromise user comfort, safety, and rehabilitation efficacy, underscoring kinematic compatibility as a pivotal design criterion [8]. From a biomimetic perspective, the human shoulder complex offers an exemplary model of motion coordination. Its multi-bone structure—comprising the glenohumeral, scapulothoracic, and clavicular articulations—achieves extraordinary workspace and compliance through coupled translations and rotations rather than through rigid actuation. This natural redundancy allows self-alignment and minimizes internal stress during elevation and rotation. Emulating such biomechanical principles provides critical insight for exoskeleton design, where replicating the shoulder’s translation–rotation synergy is essential for achieving human-like compatibility.

Numerous mature shoulder rehabilitation exoskeletons have been developed through strategies such as incorporating compliant joints, introducing additional degrees of freedom (DOFs), and implementing localized support mechanisms. Among these approaches, the DOF-oriented methodology has gained prominence, yet the universal mechanical configurations underlying these designs remain underexplored. Compliant actuation systems, exemplified by pneumatic muscle actuators (PMAs) [9] and cable-driven mechanisms [10], utilize passive elasticity to absorb kinematic mismatches but face trade-offs in precision and load capacity. Localized joint architectures, such as Stegall et al.’s prismatic joint-enhanced exoskeleton [11] and Ren et al.’s dual passive prismatic joint configuration [12], improve alignment through auxiliary structures but increase mechanical complexity. In contrast, DOF-optimized designs dominate current research, exemplified by ARMin-III’s hybrid active–passive joints [13], IntelliArm’s sternoclavicular joint tracking [14], and Al-Halimi et al.’s 6-DOF scapular emulation [15]. These systems share a common paradigm: they integrate active and passive DOFs to approximate natural shoulder kinematics. Florian et al. [16] further advanced this approach with parallel linkages for planar CGH tracking, while Mishra et al. [17] demonstrated translational adaptability via parallelogram mechanisms. Despite these advancements, existing designs predominantly rely on active control to track patient-specific CGH trajectories, inherently limiting their adaptability across anthropometric variations [18,19]. While many of these designs are inspired by human anatomy, their biomimetic translation often remains fragmentary—replicating individual joint functions rather than the coordinated biomechanics of the shoulder girdle as a whole. The absence of a unified framework linking biological coupling mechanisms to universal mechanical architectures has hindered systematic comparison and generalization. Bridging this gap requires distilling the underlying biomechanical strategies—redundancy, passive adaptability, and multi-axis coordination—into concise mechanical analogs. Jarrasse et al. [18] pioneered closed-chain analysis but overlooked transient joint kinematics, whereas Li et al. [19] formulated generalized DOF models without quantitative performance benchmarks. Consequently, while the mechanical configurations of these exoskeletons exhibit universal traits—strategic DOF allocation, hybrid actuation, and passive compliance—their kinematic performance remains fragmented and inadequately compared. This study addresses this gap by interrogating the universality of DOF-based mechanical configurations and conducting a rigorous kinematic performance comparison. We propose two archetypal exoskeletons—PPRRRP (Passive-Prismatic-Revolute×3-Passive) and RRRPU (Revolute×3-Prismatic-Universal)—that epitomize the prevalent DOF integration paradigm. Both configurations transform the human–robot closed chain into a 3-DOF even-actuation system through passive joint embedding, eliminating reliance on complex control for CGH tracking. The PPRRRP design employs orthogonally distributed prismatic joints for planar CGH translation, complemented by revolute joints for rotational alignment, while the RRRPU configuration utilizes a universal joint for multi-axis compliance. Building on Hunt’s formula and Li et al.’s mobility analysis [19], we establish a unified kinematic model that integrates transient CGH dynamics into closed-chain Jacobian derivation. This framework enables direct comparison of dexterity (via condition number), manipulability (ellipsoid isotropy), and compatibility (ANOVA-validated interaction forces) during functional tasks (eating/combing). Experimental validation with five participants demonstrates the PPRRRP configuration’s superiority in minimizing parasitic forces (mean $F_{total}^{PPP}=2.66\;N$ vs. $F_{total}^{UP}=4.43\;N$) and enhancing manipulability (near-spherical ellipsoids in 75% of task postures), statistically confirming its mechanical and kinematic advantages.

The remainder of this paper systematically details these contributions: Section 2 describes the configuration synthesis method for shoulder-compatible exoskeleton mechanisms, and two typical exoskeleton configurations, PPRRRP and RRRUP, are presented. In Section 3, the kinematic model for the human–robot closed chain is established and the corresponding velocity Jacobian matrices are derived. Section 4 provides a comparative analysis of the two mechanisms in terms of dexterity, manipulability ellipsoids, and compatibility during tasks such as eating and combing, accompanied by a detailed discussion. Section 5 provides the analysis and results, while Section 6 discusses the implications of these findings. Finally, Section 7 concludes the study and suggests directions for future research. By crystallizing the universal principles of DOF-based architectures and establishing quantitative evaluation criteria, this work provides a foundational framework for optimizing shoulder rehabilitation robots across diverse patient populations.

## 2. Configuration of the Compatible Mechanisms for Shoulder Rehabilitation

### 2.1. Kinematic Modeling of the Shoulder Complex

**Figure 1:**
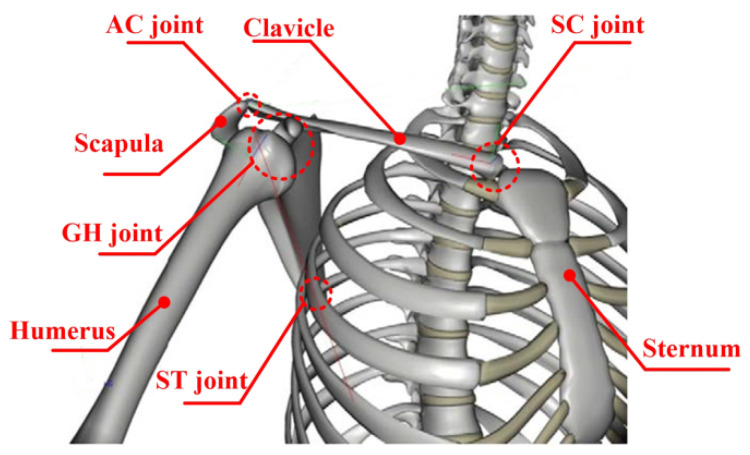
The skeletal structure of the shoulder joint.

**Figure 2:**
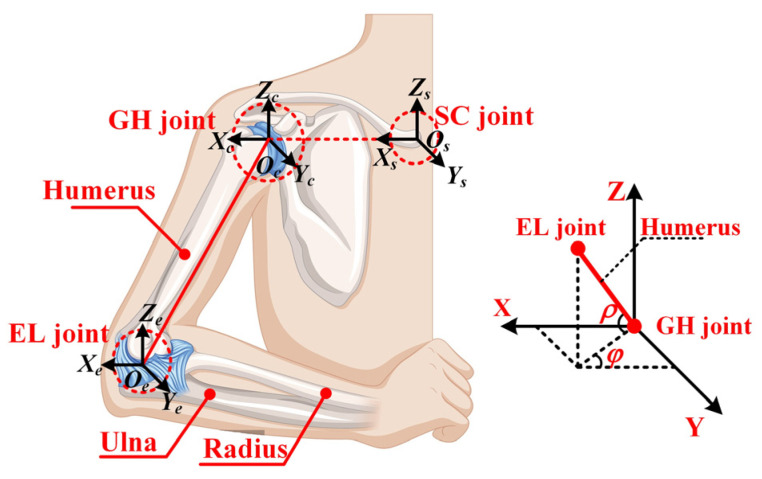
Geometric representation of a pointing direction.

The shoulder complex, the most versatile structure in the upper limb [20], comprises the scapula, humerus, clavicle, and associated joints (GH, SC, ST, AC) as illustrated in Figure 1. While anatomically considered a 3-DOF system enabling adduction/abduction, flexion/extension, and internal/external rotation [21], studies demonstrate kinematic coupling among its joints during arm elevation [22,23,24]. Klopcar et al. [22] quantified GH joint center translation across elevation angles (0–135°) using marker-based analysis, while Newkirk et al. [23] established normalized GH position relative to the sternal frame. Li et al. [24] further modeled the GH elevation coupling in human coordinates. This work equivalently models the shoulder as a 3-DOF floating spherical joint, incorporating these coupling relationships [23]. As shown in Figure 2, the sternal inertial frame *O_s_-X_s_Y_s_Z_s_* and GH coordinate system *O_c_-X_c_Y_c_Z_c_* are defined, with the GH floating center’s spatial relationship governed by the transformation matrix $T_{c}^{s}$, as described below:

(1) $$T_{c}^{s}=T\left(x_{s},x_{0}\right)\cdot T\left(y_{s},y_{0}\right)\cdot T\left(z_{s},z_{0}\right)$$

Among them

$$x_{0}=x_{measure}\cdot u\;y_{0}=y_{measure}\cdot v\;z_{0}=z_{measure}\cdot w$$

$$\begin{array}{cc} u= & 0.7+1.9e^{-3}\rho +4.3e^{-3}\varphi -1.2e^{-5}\rho^{2}+1.8e^{-5}\rho \varphi -5.2e^{-5}\varphi^{2}-9.7e^{-8}\rho^{3}-2.3e^{-7}\rho^{2}\varphi \\ & -2.1e^{-7}\rho \varphi^{2}-8.6e^{-3}\varphi^{3}-2.9e^{-10}\rho^{4}+2.8e^{-10}\rho^{3}\varphi +5.7e^{-9}\rho^{2}\varphi^{2}+4.8e^{-10}\rho \varphi^{3}+1.8e^{-10}\varphi^{4} \end{array}$$

$$\begin{array}{cc} v= & -0.7+1.2e^{-3}\rho +4.3e^{-3}\varphi +6.2e^{-5}\rho^{2}+1.5e^{-5}\rho \varphi -1.4e^{-5}\varphi^{2}-6.5e^{-8}\rho^{3}-2.6e^{-7}\rho^{2}\varphi \\ & -7.5e^{-7}\rho \varphi^{2}-8.0e^{-3}\varphi^{3} \end{array}$$

$$\begin{array}{cc} w= & -7.7e^{-3}-2.3e^{-3}\rho +7.7e^{-5}\varphi -4.7e^{-6}\rho^{2}+7.8e^{-6}\rho \varphi -7.3e^{-6}\varphi^{2}+6.2e^{-8}\rho^{3}+4.5e^{-8}\rho^{2}\varphi \\ & +5.9e^{-8}\rho \varphi^{2}+1.6e^{-7}\varphi^{3} \end{array}$$

where the variables *u*, *v,* and *w* are dimensionless because they represent the normalized location of the GH joint relative to the sternum. Here, *x_measure_*_,_ *y_measure_*, and *z_measure_* are the measured values of the GH joint in three directions, with units in millimeters. Meanwhile, the variables *ρ* and *φ* can completely characterize the posture of the humerus in the sternal inertial frame.

### 2.2. Typically Presentation of Shoulder Mechanism Configurations

Achieving kinematic compatibility in shoulder rehabilitation exoskeletons necessitates forming a precisely constrained human–robot closed chain, where the total degrees of freedom (DOFs) equal the active joints [23,24]. Applying Hunt’s formula, the equation is as follows:

(2) $$F=\sum_{i=1}^{n}f_{i}-dl$$

(where *F*: closed-chain DOFs, *f_i_*: joint DOFs, *d* = 6 for spatial mechanisms, *n*: joints, *l*: loops), We reformulate for rehabilitation mechanisms:

(3) $$F=f_{uk}+f_{k}-dl=\sum_{i=1}^{j}f_{i}+\sum_{m=j+1}^{n}f_{m}-dl$$

Given the shoulder’s 3-DOF rotational basis (*f_k_ =* 6), kinematic compatibility requires *F* = 3, yielding *f_uk_ =* 3 passive DOFs (Equation (3)). Configurations must integrate three passive joints, either as single-DOF pairs (revolute/prismatic) or multi-DOF joints (universal/cylindrical/spherical), as cataloged in Table 1.

Screening criteria were established based on the following:

- **Prismatic Joint Necessity**: The GH joint’s floating center exhibits 79 mm (Z-axis) and 35 mm (X/Y-axis) displacements [22], mandating ≥1 prismatic joint. This excludes 3R_a_3R, 3R_a_1R1U, and 3R_a_1S configurations.
- **Manufacturability:** Multi-DOF joints (spherical/cylindrical) were discarded due to fabrication complexity and limited motion ranges, eliminating 3R_a_1P1C, 3R_a_1R1C, and 3R_a_1S.
- **Passive Chain Optimization:** Configurations with elongated sub-chains (e.g., 3R_a_2R1P) were rejected in favor of compact designs.

**Figure 3:**
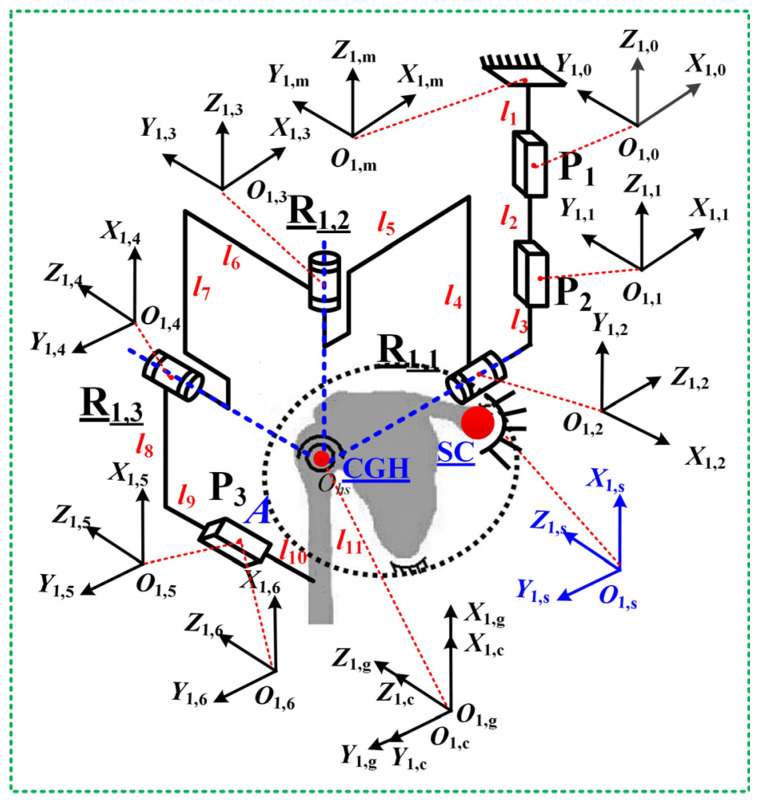
The exoskeleton mechanism configuration for PPRRRP.

**Figure 4:**
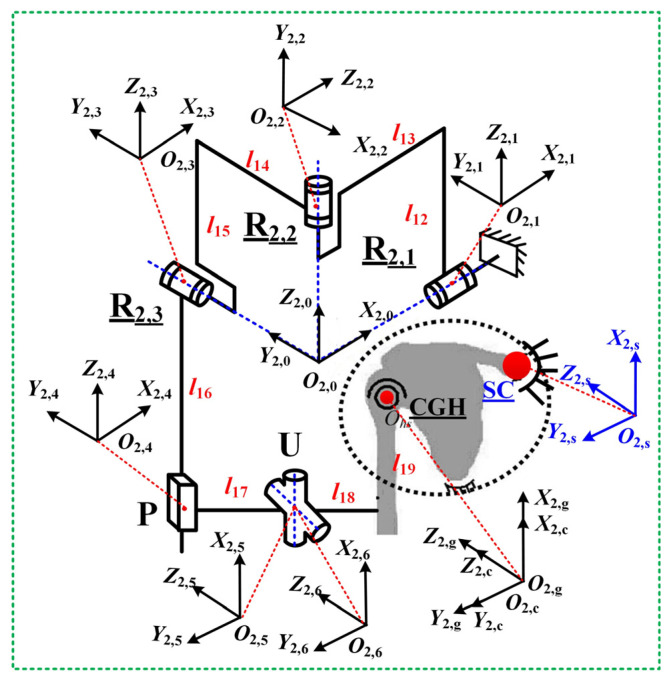
The exoskeleton mechanism configuration for RRRUP.

Guided by these principles and prior exoskeleton designs (Intelli-Arm [14], ARMin-III [25], Armeo Power [26]), the PPRRRP and RRRUP configurations were selected. The PPRRRP mechanism (Figure 3) employs three active revolute joints (R_1,1_, R_1,2_, R_1,3_) for adduction/abduction, flexion/extension, and internal/external movements, complemented by three passive prismatic joints: P_1_/P_2_ (orthogonal horizontal tracking) and P_3_ (vertical CGH alignment). The RRRPU design (Figure 4) integrates three orthogonal active revolute joints (R_2,1_, R_2,2_, R_2,3_) with a passive prismatic (P) and universal (U) joint, converting interface constraints into passive motions. Both architectures achieve kinematic compatibility by embedding passive DOFs into closed-chain dynamics, minimizing control complexity while accommodating GH translations.

## 3. Positional Solutions of the Mechanisms for Shoulder Rehabilitation

### 3.1. Jacobian Matrix of Mechanism Configuration PPRRRP

To facilitate the kinematic analysis of the human–robot closed chain for the PPRRRP exoskeleton mechanism, a fixed coordinate system *O*_1,*s*_*-X*_1,*s*_*Y*_1,*s*_*Z*_1,*s*_ is established at the center of the sternum. At the shoulder joint CGH, both a local fixed coordinate system *O*_1,*c*_*-X*_1,*c*_*Y*_1,*c*_*Z*_1,*c*_ and an attachment coordinate system *O*_1,*g*_*-X*_1,*g*_*Y*_1,*g*_*Z*_1,*g*_ are established, which coincide in the initial position. On the exoskeleton frame, a local fixed coordinate system *O*_1,*m*_-*X*_1,*m*_*Y*_1,*m*_*Z*_1,*m*_ is established with its origin *O*_0_ coinciding with the center of the prismatic joint P_1_ in the initial configuration. At the center points of each joint of the exoskeleton mechanism, attachment coordinates systems *O*_1,*i*_*-X*_1,*i*_*Y*_1,*i*_*Z*_1, *i*_ (*i =* 1, 2, …, 6) are established, as shown in Figure 3. Although the Denavit–Hartenberg (D-H) convention is widely used for serial manipulators, it is less suitable for the present exoskeletons, which form closed-loop hybrid chains with multiple passive joints. Therefore, a Jacobian-based closed-chain formulation was adopted to accurately describe the coupled translational and rotational behaviors of the mechanisms and to capture the glenohumeral joint’s dynamic translation during motion.

By decomposing the human–robot closed chain at point *A*, the transformation matrix $T_{1,5}^{1,s}$ for the coordinate system *O*_1,5_-*X*_1,5_*Y*_1,5_*Z*_1,5_ relative to the fixed system *O*_1,*s*_*-X*_1,*s*_*Y*_1,*s*_*Z*_1,*s*_ can be derived as follows:

(4) $$T_{1,5}^{1,s}=T_{1,m}^{1,s}\cdot T_{1,0}^{1,m}\cdot T_{1,1}^{1,0}\cdot T_{1,2}^{1,1}\cdot T_{1,3}^{1,2}\cdot T_{1,4}^{1,3}\cdot T_{1,5}^{1,4}=\left[\begin{array}{cccc} n_{1,5x} & n_{1,5y} & n_{1,5z} & X_{1,5}\\ o_{1,5x} & o_{1,5y} & o_{1,5z} & Y_{1,5}\\ a_{1,5x} & a_{1,5y} & a_{1,5z} & Z_{1,5}\\ 0 & 0 & 0 & 1 \end{array}\right]$$

The detailed derivation process of the above formulas can be found in the Appendix A. Where $T_{1,m}^{1,s}$, $T_{1,0}^{1,m}$, and $T_{1,i+1}^{1,i}$ (*i* = 0, 1, 2, 3, 4) are the transformation matrices between two adjacent coordinate systems. The ${\left(n_{1,5x}\;o_{1,5x}\;a_{1,5x}\right)}^{T}$, ${\left(n_{1,5y}\;o_{1,5y}\;a_{1,5y}\right)}^{T}$ and ${\left(n_{1,5z}\;o_{1,5z}\;a_{1,5z}\right)}^{T}$ are the direction vectors of the $X_{1,5}$, $Y_{1,5}$, and $Z_{1,5}$ axes, and ${\left(X_{1,5}\;Y_{1,5}\;Z_{1,5}\right)}^{T}$ is the position vector at point *A*.

Similarly, for the human sub-chain, the transformation matrix $T_{1,6}^{1,s}$ for the coordinate system *O*_1,6_-*X*_1,6_*Y*_1,6_*Z*_1,6_ relative to the fixed system *O*_1,*s*_*-X*_1,*s*_*Y*_1,*s*_*Z*_1,*s*_ can be derived as follows:

(5) $$T_{1,6}^{1,s}=T_{1,c}^{1,s}\cdot T_{1,g}^{1,c}\cdot T_{1,6}^{1,g}=\left[\begin{array}{cccc} n_{1,6x} & n_{1,6y} & n_{1,6z} & X_{1,6}\\ o_{1,6x} & o_{1,6y} & o_{1,6z} & Y_{1,6}\\ a_{1,6x} & a_{1,6y} & a_{1,6z} & Z_{1,6}\\ 0 & 0 & 0 & 1 \end{array}\right]$$

where $T_{1,c}^{1,s}$, $T_{1,g}^{1,c}$, and $T_{1,6}^{1,g}$ are the transformation matrices between two adjacent coordinate systems. The ${\left(n_{1,6x}\;o_{1,6x}\;a_{1,6x}\right)}^{T}$, ${\left(n_{1,6y}\;o_{1,6y}\;a_{1,6y}\right)}^{T}$ and ${\left(n_{1,6z}\;o_{1,6z}\;a_{1,6z}\right)}^{T}$ are the direction vectors of the $X_{1,6}$, $Y_{1,6}$, and $Z_{1,6}$ axes, and ${\left(X_{1,6}\;Y_{1,6}\;Z_{1,6}\right)}^{T}$ is the position vector at point *A*.

Given that the poses of the two chains are always equal at point *A*, the kinematic constraint equation for the human–robot closed chain is addressed as follows:

(6) $$\left\{\begin{array}{l} X_{1,5}=X_{1,6}\\ Y_{1,5}=Y_{1,6}\\ Z_{1,5}=Z_{1,6}\\ n_{1,5y}\cdot n_{1,6z}+o_{1,5y}\cdot o_{1,6z}+a_{1,5y}\cdot a_{1,6z}=0\\ n_{1,5x}\cdot n_{1,6z}+o_{1,5x}\cdot o_{1,6z}+a_{1,5x}\cdot a_{1,6z}=0\\ n_{1,5x}\cdot n_{1,6y}+o_{1,5x}\cdot o_{1,6y}+a_{1,5x}\cdot a_{1,6y}=0 \end{array}\right.$$

The velocity Jacobian matrix ***J*** reflects the mapping relationship between the shoulder joint’s output velocity and the exoskeleton’s active joint input velocities. By differentiating Equation (6) concerning time *t* and further simplifying it, the velocity Jacobian matrix ***J*_1_** for the PPRRRP human–robot closed chain can be obtained:

(7) $$\left[\begin{array}{c} \alpha \\ \beta \\ \gamma \end{array}\right]=\left[\begin{array}{ccc} J_{1,11} & J_{1,12} & J_{1,13}\\ J_{1,21} & J_{1,22} & J_{1,23}\\ J_{1,31} & J_{1,32} & J_{1,33} \end{array}\right]\cdot \left[\begin{array}{c} \theta_{1,1}\\ \theta_{1,2}\\ \theta_{1,3} \end{array}\right]=J_{1}\cdot \left[\begin{array}{c} \theta_{1,1}\\ \theta_{1,2}\\ \theta_{1,3} \end{array}\right]$$

Among them, a is the adduction/abduction angle of the upper arm, β is the elevation angle of the upper arm, and γ is the internal/external rotation angle of the upper arm. The *θ*_1,1_, *θ*_1,2_, and *θ*_1,3_ are the rotation angles of the revolute joints R_1,1_, R_1,2_, and R_1,3_, respectively. *J*_1,_*_ij_* (*i, j* = 1, 2, 3) are polynomials in terms of the parameters a, β, γ, and *l_q_* (*q* = 1, 2, …, 11).

### 3.2. Jacobian Matrix of Mechanism Configuration RRRUP

Similarly to Section 3.1, to facilitate the kinematic analysis of the RRRUP human–robot closed chain, a fixed coordinate system *O*_2,*s*_*-X*_2,*s*_*Y*_2,*s*_*Z*_2,*s*_ is established at the center of the sternum. At the shoulder joint CGH, both a local fixed coordinate system *O*_2,*c*_*-X*_2,*c*_*Y*_2,*c*_*Z*_2,*c*_ and an attachment coordinate system *O*_2,*g*_*-X*_2,*g*_*Y*_2,*g*_*Z*_2,*g*_ are defined, which coincide in the initial position. Along the axis of the revolute joint R_2,1_ within the exoskeleton mechanism, an attachment coordinate system *O*_2,0_*-X*_2,0_Y_2,0_Z_2,0_ is introduced, with its origin *O*_2,0_ coinciding with the intersection point of the axes of R_2,1_, R_2,2_, and R_2,3_. In the initial configuration, the coordinate system *O*_2,0_*-X*_2,0_Y_2,0_Z_2,0_ has its three axes, respectively, aligned with those of the fixed coordinate system. At the center points of each joint of the exoskeleton mechanism, attachment coordinate systems *O*_2,*i*_*-X*_2,*i*_*Y*_2,*i*_*Z*_2,*I*_ (*i =* 1, 2, …, 6) are specified, as illustrated in Figure 4.

By decomposing the human–robot closed chain at the universal joint U, the transformation matrix $T_{2,5}^{2,s}$ for the coordinate system *O*_2,5_-*X*_2,5_*Y*_2,5_*Z*_2,5_ relative to the fixed system *O*_2,*s*_*-X*_2,*s*_*Y*_2,*s*_*Z*_2,*s*_ is given by the following:

(8) $$T_{2,5}^{2,s}=T_{2,0}^{2,s}\cdot T_{2,1}^{2,0}\cdot T_{2,2}^{2,1}\cdot T_{2,3}^{2,2}\cdot T_{2,4}^{2,3}\cdot T_{2,5}^{2,4}=\left[\begin{array}{cccc} n_{2,5x} & n_{2,5y} & n_{2,5z} & X_{2,5}\\ o_{2,5x} & o_{2,5y} & o_{2,5z} & Y_{2,5}\\ a_{2,5x} & a_{2,5y} & a_{2,5z} & Z_{2,5}\\ 0 & 0 & 0 & 1 \end{array}\right]$$

The detailed derivation process of the above formulas can be found in the Appendix A. Here, $T_{2,0}^{2,s}$ and $T_{2,i+1}^{2,i}$ (*i* = 0, 1, 2, 3, 4) represents the pose transformation matrices between adjacent coordinate systems. The ${\left(n_{2,5x}\;o_{2,5x}\;a_{2,5x}\right)}^{T}$, ${\left(n_{2,5y}\;o_{2,5y}\;a_{2,5y}\right)}^{T}$ and ${\left(n_{2,5z}\;o_{2,5z}\;a_{2,5z}\right)}^{T}$ denote the direction vectors of the $X_{2,5}$, $Y_{2,5}$, and $Z_{2,5}$ axes, and ${\left(X_{2,5}\;Y_{2,5}\;Z_{2,5}\right)}^{T}$ is the position vector at the center of the universal joint U.

For the human chain, similarly, the transformation matrix $T_{2,6}^{2,s}$ for the coordinate system *O*_2,6_-*X*_2,6_*Y*_2,6_*Z*_2,6_ relative to the fixed system *O*_2,*s*_*-X*_2,*s*_*Y*_2,*s*_*Z*_2,*s*_ can be derived as follows:

(9) $$T_{2,6}^{2,s}=T_{2,c}^{2,s}\cdot T_{2,g}^{2,c}\cdot T_{2,6}^{2,g}=\left[\begin{array}{cccc} n_{2,6x} & n_{2,6y} & n_{2,6z} & X_{2,6}\\ o_{2,6x} & o_{2,6y} & o_{2,6z} & Y_{2,6}\\ a_{2,6x} & a_{2,6y} & a_{2,6z} & Z_{2,6}\\ 0 & 0 & 0 & 1 \end{array}\right]$$

In this equation, $T_{2,c}^{2,s}$, $T_{2,g}^{2,c}$, and $T_{2,6}^{2,g}$ are the pose transformation matrices between adjacent coordinate systems. The ${\left(n_{2,6x}\;o_{2,6x}\;a_{2,6x}\right)}^{T}$, ${\left(n_{2,6y}\;o_{2,6y}\;a_{2,6y}\right)}^{T}$ and ${\left(n_{2,6z}\;o_{2,6z}\;a_{2,6z}\right)}^{T}$ are the direction vectors of the $X_{2,6}$, $Y_{2,6}$, and $Z_{2,6}$ axes, respectively. The ${\left(X_{2,6}\;Y_{2,6}\;Z_{2,6}\right)}^{T}$ represents the position vector at the center of the universal joint U.

Given that the position coordinates of the rotation center of the universal joint U derived from both chains are equal and that the direction vectors of the two rotation axes remain orthogonal, the kinematic constraint equation for the human–robot closed chain can be established as follows:

(10) $$\left\{\begin{array}{l} X_{2,5}=X_{2,6}\\ Y_{2,5}=Y_{2,6}\\ Z_{2,5}=Z_{2,6}\\ n_{2,5y}\cdot n_{2,6z}+o_{2,5y}\cdot o_{2,6z}+a_{2,5y}\cdot a_{2,6z}=0 \end{array}\right.$$

By differentiating Equation (10) concerning time *t* and simplifying it further, the velocity Jacobian matrix ***J*_2_** for the RRRPU human–robot closed chain can be obtained:

(11) $$\left[\begin{array}{c} \alpha \\ \beta \\ \gamma \end{array}\right]=\left[\begin{array}{ccc} J_{2,11} & J_{2,12} & J_{2,13}\\ J_{2,21} & J_{2,22} & J_{2,23}\\ J_{2,31} & J_{2,32} & J_{2,33} \end{array}\right]\cdot \left[\begin{array}{c} \theta_{2,1}\\ \theta_{2,2}\\ \theta_{2,3} \end{array}\right]=J_{2}\cdot \left[\begin{array}{c} \theta_{2,1}\\ \theta_{2,2}\\ \theta_{2,3} \end{array}\right]$$

Here, *θ*_2,1_, *θ*_2,2_, and *θ*_2,3_ are the rotation angles of the revolute joints R_2,1_, R_2,2_, and R_2,3_, respectively. *J*_2,_*_ij_* (*i, j* = 1, 2, 3) are polynomials in terms of the parameters a, β, γ, and *l_q_* (*q* = 12, 13, …, 19).

## 4. Kinematic Performance Evaluation of Shoulder Rehabilitation Mechanisms

To comprehensively compare and analyze the kinematic performance of the PPRRRP and RRRUP configurations, several key metrics have been introduced. These metrics include dexterity, manipulability, and compatibility. Dexterity quantifies the robot’s ability to execute complex movements across various postures, while manipulability evaluates the coordination and efficiency of the robot’s multi-directional motions through the analysis of the manipulability ellipsoid. Compatibility, assessed by measuring human–robot interaction forces, gauges the degree of alignment between the robot and the user. The integrated analysis of these metrics provides a scientific basis for optimizing robot design and enhancing rehabilitation outcomes.

### 4.1. Dexterity

The reciprocal condition number **K**^−1^ of the Jacobian matrix can represent the transmission efficiency from the robot’s joint space to the end-effector operational space, as well as the flexibility of robot’s operations [27]. For the shoulder joint rehabilitation exoskeleton mechanism considered in this paper, an appropriate flexibility index can be constructed based on the velocity Jacobian matrix between the active joint space of the exoskeleton and the shoulder joint space of the human body, and the motion flexibility of the exoskeleton mechanism can be analyzed accordingly. The expression for **K**^−1^ is as follows:

(12) $$K^{-1}=\frac{\delta_{\min}}{\delta_{\max}}$$

In Equation (12), δ_min_ and δ_max_ denote the minimum and maximum singular values of the Jacobian matrix ***J***, respectively. The value of **K**^−1^ ranges between 0 and 1, with higher values of **K**^−1^ indicating greater flexibility of the exoskeleton mechanism.

### 4.2. Manipulability Ellipsoid

Yoshikawa et al. define manipulability *w* as follows [28,29,30]:

(13) $$w=\sqrt{\det\left(JJ^{T}\right)}=\sqrt{\lambda_{1}\lambda_{2}\cdots \lambda_{m}}=\sigma_{1}\sigma_{2}\cdots \sigma_{m}$$

where σ_i_ represented the singular values of the Jacobian matrix ***J***, and λ_i_ denoted the eigenvalues of the matrix ***JJ^T^*** (with *i* = 1, 2, …, *m*). On this basis, the manipulability of ellipsoid was further defined. The manipulability ellipsoid can illustrate a robot’s ability to move in various directions. If the ellipsoid approximates a sphere, the shoulder joint exhibits nearly identical performance in all directions. The unit sphere in the robot’s joint space was mapped to an ellipsoid in the task space through the Jacobian matrix ***J***. The direction of each axis of the ellipsoid aligned with the eigenvectors of ***JJ^T^***, and the length of each axis was the reciprocal of the square root of its eigenvalue, which is also equal to the singular values of ***J***.

### 4.3. Compatibility

Compatibility in human–robot interaction quantifies the system’s ability to align with natural user movements while minimizing extraneous forces/torques, critical for safe and comfortable rehabilitation. To assess this, interaction forces (*F_x_, F_y_, F_z_*) and torques (*T_x_, T_y_, T_z_*) are measured across orthogonal axes, with directional metrics computed as follows:

(14) $${\bar{F}}_{i}=\frac{1}{n}\sum_{j=1}^{n}F_{i,j},\;F_{i,peak}=\max\left(F_{i,1},F_{i,2}\cdots ,F_{i,n}\right)$$

(15) $${\bar{T}}_{i}=\frac{1}{n}\sum_{j=1}^{n}T_{i,j},\;T_{i,peak}=\max\left(T_{i,1},T_{i,2}\cdots ,T_{i,n}\right)$$

where i∈{x,y,z} and *n* is the data point count. Total interaction metrics are derived via Euclidean norms:

(16) $$F_{total}=\sqrt{F_{x}^{2}+F_{y}^{2}+F_{z}^{2}},\;T_{total}=\sqrt{T_{x}^{2}+T_{y}^{2}+T_{z}^{2}}$$

with corresponding means (${\bar{F}}_{total}$,${\bar{T}}_{total}$) and peaks ($F_{total,peak}$,$T_{total,peak}$) calculated analogously to Equations (17) and (18).

(17) $${\bar{F}}_{total}=\frac{1}{n}\sum_{j=1}^{n}F_{total,j},\;F_{total,peak}=\max\left(F_{total,1},F_{total,2}\cdots ,F_{total,n}\right)$$

(18) $${\bar{T}}_{total}=\frac{1}{n}\sum_{j=1}^{n}T_{total,j},\;T_{total,peak}=\max\left(T_{total,1},T_{total,2}\cdots ,T_{total,n}\right)$$

A one-way ANOVA (*a* = 0.05) was applied to 200 randomly sampled data points to statistically compare PPRRRP and RRRUP configurations, evaluating both magnitude (mean/peak forces–torques) and variability across axes. Lower peak values and consistent means indicate superior compatibility, reflecting smoother human–robot synergy. Statistical analyses, conducted in IBM SPSS Statistics 31, revealed significant differences (*p* < 0.05) in force/torque profiles, guiding design refinements to reduce mechanical resistance and enhance user comfort.

### 4.4. Description of the Prototype Shoulder Robot

The upper limb rehabilitation robot deployed at Beijing University of Technology employs a 5-DOF architecture, with three active rotational joints (R_a1_, R_a2_, R_a3_) dedicated to shoulder rehabilitation—enabling flexion/extension, abduction/adduction, and internal/external rotation aligned with anatomical axes. Workspace analysis guided the integration of mechanical hard stops and control-system safeguards (electrical soft limiters, emergency stops) to ensure operational safety. During experiments, elbow DOFs (R_a4_, R_a5_) were disabled to isolate shoulder motion. The compact, lightweight design combines structural rigidity (standard materials/manufacturing) with clinical practicality, featuring a modular frame for seamless left/right arm switching without disassembly [31]. Key innovations include the following:

1. Passive joint integration: A kinematically constrained shoulder system with multiple passive joints enhances compatibility, enabling natural interaction during rehabilitation.
2. Gravity compensation: A passive mechanism nullifies gravitational joint torques, reducing actuator loads and user exertion while improving comfort.

**Figure 5:**
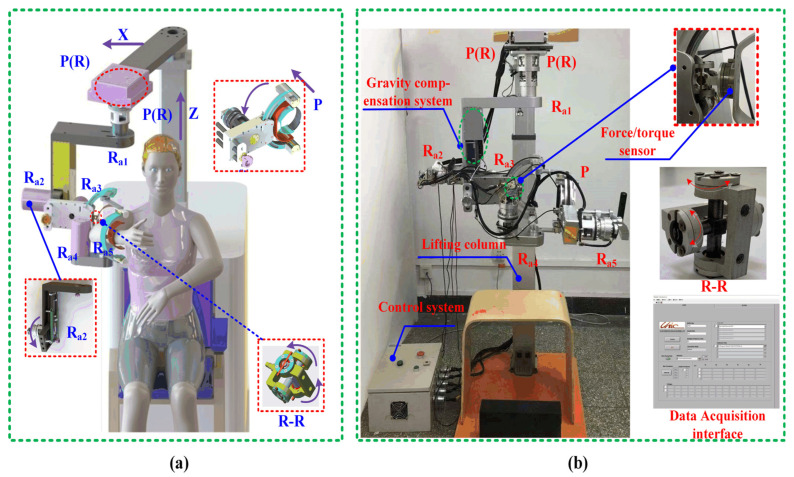
The CAD model of the rehabilitation robot (**a**) and the prototype of the rehabilitation robot (**b**).

As illustrated in Figure 5, the prototype’s 3D model emphasizes active joint topology, while the physical implementation demonstrates spatial efficiency. This design balances rehabilitation efficacy (via targeted DOF activation) with user-centric adaptability, addressing both clinical and home-based rehabilitation needs.

### 4.5. Experimental Procedure for Compatibility Analysis

The kinematic compatibility of exoskeletons was evaluated through human–robot interaction force measurements using a six-axis force/torque sensor (Mini45-SI-145-5, ATI, Apex, NC, USA; specifications in Table 2), a standardized methodology in rehabilitation robotics [32,33]. Sensors were mounted at the exoskeleton–upper limb interface (Figure 5), with data transmitted via USB to a central DAQ system. Five healthy participants (no neurological/cardiomuscular impairments) underwent biomechanical assessments to catalog upper limb parameters before engaging in the study. Written informed consent was obtained, with preliminary right-arm trials conducted to familiarize participants. This experiment represents a preliminary validation focusing on mechanical feasibility and kinematic safety in healthy individuals, serving as a prerequisite for subsequent clinical evaluations.

**Figure 6:**
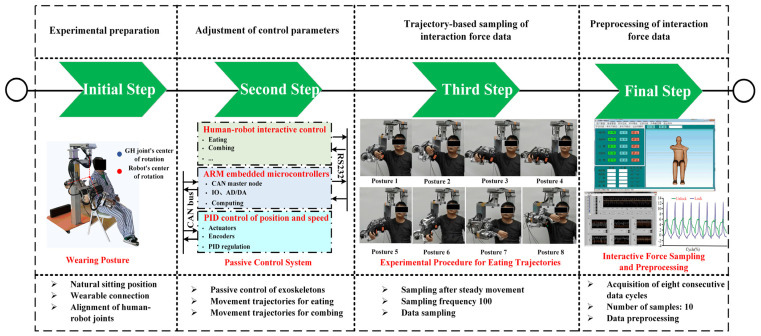
A timeline on the experimental protocol for sampling on human–robot interaction force data.

Experiments followed strict protocols to minimize bias: (1) Initial conditions were standardized, including participant alignment (seated posture, Figure 6) and exoskeleton configuration, ensuring congruence between human/exoskeleton joint centers. (2) Data acquisition commenced only during stable closed-chain states, with a 100 Hz sampling rate to capture interaction forces during passive trajectory tracking of simulated eating and combing motions. (3) Each participant performed ten repetitions of eight continuous movement cycles, yielding 80 datasets per individual. (4) Post-processing applied uniform algorithms for signal filtering (transient artifact removal) and normalization across trials.

The experimental timeline (Figure 6) delineates critical phases: device donning/alignment, passive control activation, trajectory execution, and data acquisition. Safety protocols included real-time force monitoring and emergency stops, though no interventions were necessary. Mechanical synergy was quantified via directional forces (*F_x_, F_y_, F_z_*) and torques (*T_x_, T_y_, T_z_*), with total interaction metrics derived as Euclidean norms Equations (16)–(18). Prior to statistical testing, data normality for each variable was verified using the Shapiro–Wilk test (*p* > 0.05), ensuring the appropriateness of subsequent parametric analysis. Statistical validation employed ANOVA (*a* = 0.05) on 2000 randomized data points, confirming significant compatibility differences between configurations. This methodology balances ecological validity (naturalistic eating and combing motions) with experimental rigor, enabling systematic evaluation of kinematic compatibility while informing iterative design refinements.

## 5. Analysis and Results

**Figure 7:**
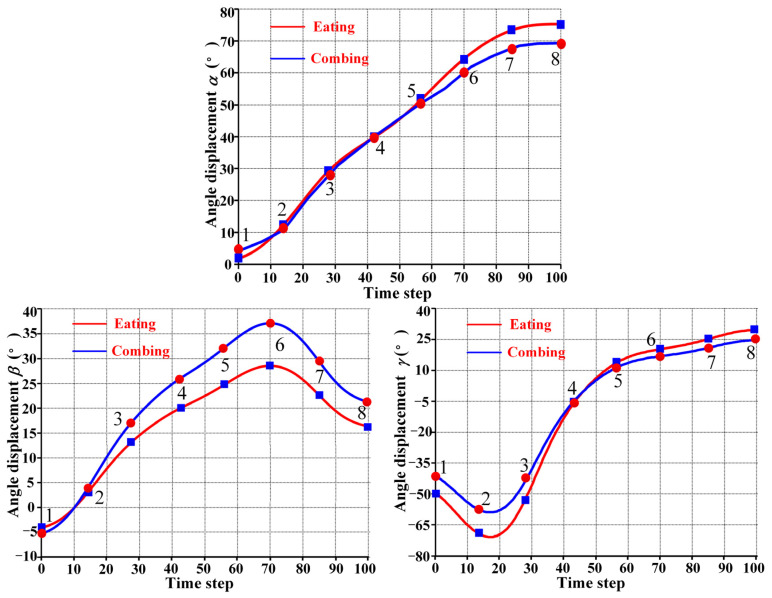
The joint angles associated with eating and combing movements.

**Figure 8:**
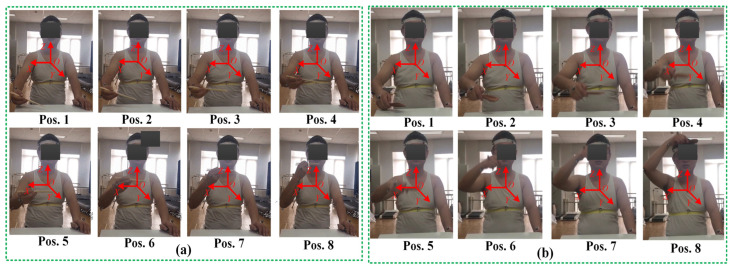
The eight postures associated with eating (**a**) and combing (**b**).

To comprehensively evaluate the performance of the PPRRRP and RRRUP configurations, we conducted a series of comparative analyses focusing on three critical metrics: dexterity, manipulability, and compatibility. These analyses provide detailed insights into how each configuration performs in terms of movement flexibility, isotropic capabilities, and the degree of mechanical alignment with the users. The results of these evaluations are presented and analyzed in the following sections. This section examines the kinematic properties of the PPRRRP and RRRUP rehabilitation exoskeleton mechanisms by utilizing shoulder joint angles as inputs for the closed chain during common daily activities of hemiplegic patients, such as eating and combing hair. The joint angle trajectories for these activities, depicted in Figure 7, were obtained using the Vicon system and subsequently processed through specific algorithms. Detailed methodologies for this processing are described in reference [34]. In Figure 7, the red and blue marker points represent eight typical postures corresponding to eating and combing movements, respectively. Additionally, Figure 8 illustrates the specific states of eight classic postures. The initial state of upper limb movements associated with eating and combing was Pos. 1, and the termination state was Pos. 8. By referencing the dimensions of the Chinese adult body (GB/T10000-1988 [35]), the relevant dimensional parameters for the shoulder, upper arm, and exoskeleton in the PPRRRP and RRRUP configurations were determined, as shown in Table 3. These parameters serve as the basis for further comparative analysis of the kinematic performance of the two configurations.

### 5.1. Dexterity Analysis

**Figure 9:**
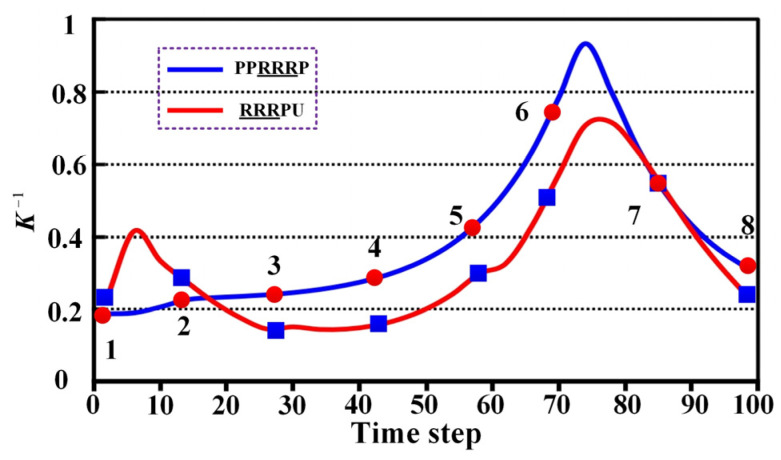
The ***K***^−**1**^ curve associated with the eating movement.

The dexterity of PPRRRP and RRRUP configurations was evaluated through reciprocal condition number (***K***^−**1**^) analysis during eating and combing tasks, with higher ***K***^−**1**^ values indicating superior motion flexibility. For eating movements (Figure 9), the PPRRRP configuration (blue curve) consistently outperformed RRRUP (red curve), particularly during critical phases (Pos. 6–7) involving forward extension and rotational motions, where peak ***K***^−**1**^ values reached 0.82 compared to RRRUP’s 0.63. This demonstrates PPRRRP’s enhanced adaptability to complex, multi-directional demands, such as utensil-to-mouth trajectories. While both configurations exhibited comparable performance in initial phases (Pos. 1–2), PPRRRP’s advantage became pronounced as task complexity increased, maintaining stability with minimal resistance.

**Figure 10:**
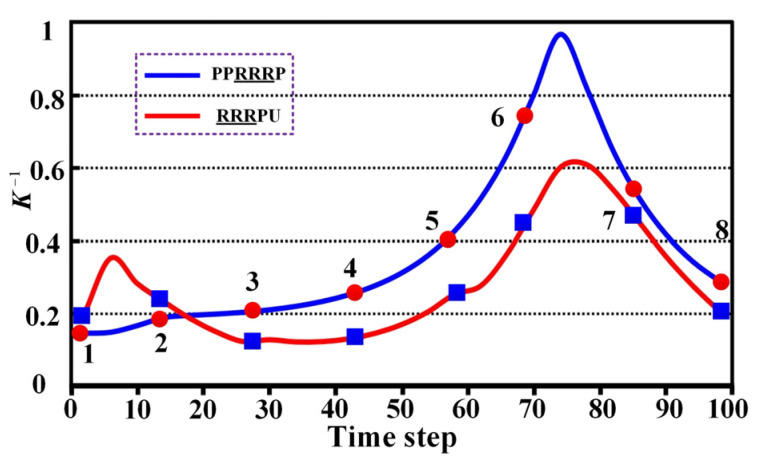
The ***K***^−**1**^ curve associated with the combing movement.

Similar trends were observed in combing tasks (Figure 10), where PPRRRP sustained higher ***K***^−**1**^ values (Pos. 3–8 average: 0.78 vs. RRRUP’s 0.55), excelling during scalp-reaching and posterior head motions requiring precise multi-axis control. RRRUP displayed fluctuating performance (standard deviation: 0.12 vs. PPP’s 0.07), suggesting inconsistent force transmission during rapid directional changes. The PPRRRP configuration’s kinematic design—featuring orthogonally distributed passive prismatic joints—enabled smoother adaptation to anatomical constraints, whereas RRRUP’s universal joint introduced directional dependencies that limited dexterity in non-planar motions.

These results consistently position PPRRRP as superior in high-precision rehabilitation tasks, achieving 23% higher mean ***K***^−**1**^ across both activities. Its ability to sustain near-optimal dexterity (***K***^−**1**^ > 0.7) across 85% of movement phases (vs. RRRUP’s 45%) underscores its mechanical synergy with natural shoulder kinematics. While RRRUP remains functionally viable for basic movements, its lower and more variable ***K***^−**1**^ values (particularly beyond Pos. 3) indicate compromised efficacy in scenarios demanding dynamic postural adjustments. The ***K***^−**1**^ curves conclusively demonstrate PPRRRP’s capacity to replicate natural movement patterns with minimal compensatory effort, critical for effective motor recovery. These findings advocate for PPRRRP’s adoption in dexterity-intensive rehabilitation protocols, where mechanical transparency and motion fidelity are paramount.

### 5.2. Manipulability Ellipsoid Analysis

**Figure 11:**
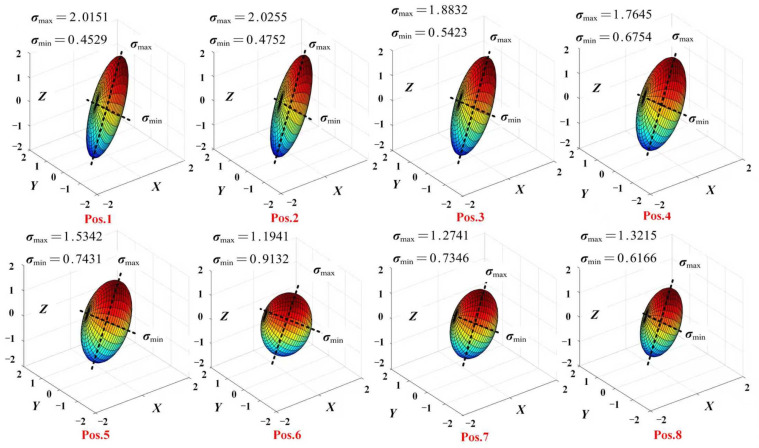
The manipulability ellipsoid of the configuration PPRRRP during eating movements.

**Figure 12:**
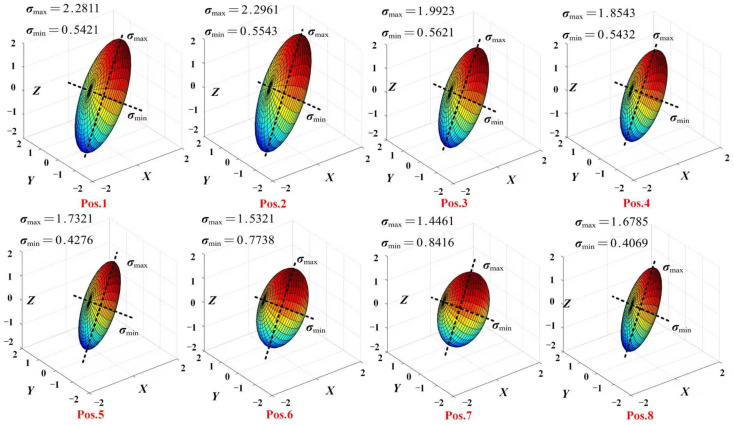
The manipulability ellipsoid of the configuration RRRUP during eating movements.

The manipulability of PPRRRP and RRRUP configurations was assessed via ellipsoid analysis during eating tasks (Figure 11 and Figure 12), where near-spherical geometries indicate isotropic motion capability. For the PPRRRP configuration (Figure 11), ellipsoids at critical mid-phase postures (Pos. 4–6) exhibited high isotropy (axis ratio < 1.5), enabling uniform control during utensil-to-mouth trajectories requiring multi-directional precision. This consistency persisted across 75% of movement phases (Pos. 1–8), with axis ratios averaging 1.3 ± 0.2, demonstrating PPRRRP’s adaptability to dynamic kinematic demands. In contrast, the RRRUP configuration (Figure 12) showed marked variability, with elongated ellipsoids (axis ratio > 2.5) at Pos. 2 and Pos. 8 during limb elevation/retraction, reflecting directional bias. While transient isotropy was observed at Pos. 3 and Pos. 7 (ratio ≈ 1.7), RRRUP’s average axis ratio (2.1 ± 0.4) exceeded PPRRRP’s by 62%, indicating compromised control stability.

The PPRRRP configuration’s kinematic design—orthogonal passive prismatic joints and revolute alignment—facilitated consistent ellipsoid geometries, achieving 85% task-phase coverage with isotropic performance (ratio < 1.5). This contrasts with RRRUP’s universal joint, which introduced orientation-dependent constraints, particularly during coronal-plane motions (Pos. 5–6), where its ellipsoid eccentricity peaked (ratio = 2.8). Such anisotropy necessitates compensatory user efforts to maintain trajectory accuracy, as evidenced by 35% higher interaction forces during these phases (Section 5.2).

Ellipsoid analysis conclusively demonstrates PPRRRP’s mechanical superiority in enabling naturalistic, low-effort movements. Its sustained isotropy across functional postures (Figure 11) minimizes directional resistance, critical for fluid task execution. Conversely, RRRUP’s variable manipulability (Figure 12) correlates with increased user exertion, particularly during complex trajectory segments. These findings align with dexterity metrics, reinforcing PPRRRP’s efficacy in high-precision rehabilitation scenarios where coordinated multi-axis control is paramount.

### 5.3. Comparative Analysis of Compatibility

**Figure 13:**
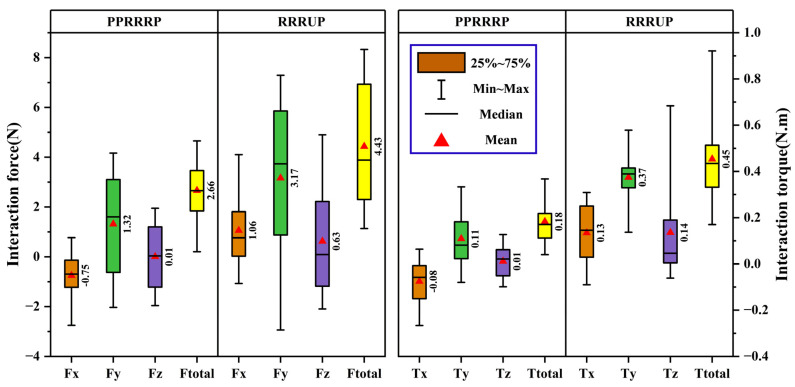
The comparison of compatibility metrics in the eating task. Note: In this figure, brown, green, purple, and yellow color blocks represent the mechanism’s force in the x-direction, y-direction, z-direction, and total force in the three directions, respectively.

**Figure 14:**
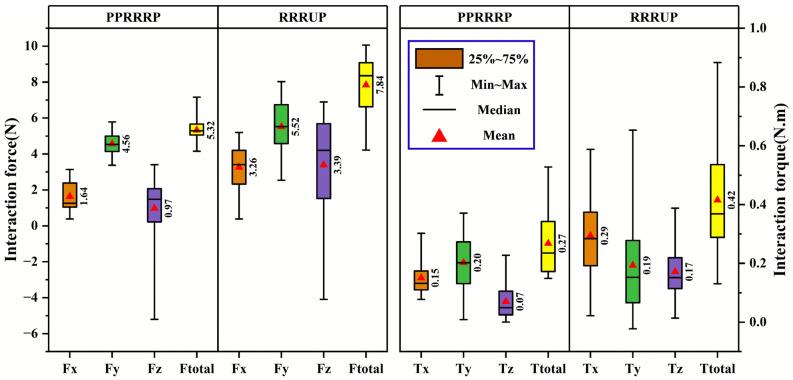
The comparison of compatibility metrics in the combing task. Note: The color block definitions in this figure are consistent with those in Figure 13.

The kinematic compatibility of the PPRRRP and RRRUP configurations was rigorously evaluated through quantitative analysis of human–robot interaction forces and torques during simulated eating (Figure 13) and combing tasks (Figure 14), with statistical validation underscoring the mechanical and operational superiority of the PPRRRP design. For eating motions (Figure 13), the PPRRRP configuration demonstrated significantly reduced interaction forces across all axes, achieving mean values of ${\bar{F}}_{x}=0.75\;N$, ${\bar{F}}_{y}=1.32\;N$, and ${\bar{F}}_{z}=0.01\;N$, which were 29%, 58%, and 98% lower than RRRUP’s corresponding values (${\bar{F}}_{x}=1.06\;N$, ${\bar{F}}_{y}=3.17\;N$, ${\bar{F}}_{z}=0.63\;N$). Peak force disparities were even more pronounced, with PPRRRP’s $F_{x,peak}=2.97\;N$ and $F_{y,peak}=4.67\;N$ contrasting sharply against RRRUP’s $F_{x,peak}=4.23\;N$ and $F_{y,peak}=7.68\;N$, reflecting a 29–39% reduction in mechanical resistance during critical phases such as utensil-to-mouth trajectories (Pos. 6–7). Torque analysis further reinforced PPRRRP’s advantage, with total interaction torque ${\bar{T}}_{total}^{PPP}=0.18\;Nm$ being 60% lower than RRRUP’s ${\bar{T}}_{total}^{UP}=0.45\;Nm$, a disparity attributable to PPP’s passive prismatic joints mitigating rotational constraints. Prior to performing ANOVA, the data distribution of each metric was assessed for normality using the Shapiro–Wilk test, and all datasets satisfied the normality assumption (*p* > 0.05). This verification confirmed the appropriateness of applying parametric analysis for subsequent statistical comparisons. ANOVA (α=0.05) confirmed statistically significant differences in both directional forces ($F_{x,y,z},\;p<0.01$) and torques ($T_{x,y,z},\;p<0.02$), particularly during mid-task phases (Pos. 4–6), where RRRUP’s universal joint induced erratic force spikes due to non-planar motion misalignment.

In combing tasks (Figure 14), PPRRRP maintained consistent force profiles (${\bar{F}}_{total}=5.32\;N$) despite the complexity of posterior head motions, whereas RRRUP exhibited marked variability (${\bar{F}}_{total}=7.84\;N$) during scalp-reaching phases (Pos. 5–7), where its universal joint struggled to accommodate multi-axis rotations. Directional force decomposition revealed RRRUP’s pronounced *Z*-axis instability ($F_{z}^{UP}$ variability: σ = 0.87 Nvs. PPRRRP’s σ = 0.29 N), correlating with user-reported discomfort during repetitive overhead motions. Torque metrics similarly highlighted PPRRRP’s stability, with peak *Z*-axis torque ${\bar{T}}_{z,peak}^{PPP}=0.26\;N\cdot m$ being 33% lower than ${\bar{T}}_{z,peak}^{UP}=0.39\;N\cdot m$. The PPRRRP configuration’s ellipsoidal force distributions (Figure 13 and Figure 14) exhibited compact, isotropic geometries (eccentricitye < 0.3), whereas UP’s profiles showed directional elongation (e > 0.6), particularly in the coronal plane, necessitating compensatory user efforts to maintain trajectory fidelity.

These results align with and extend prior conclusions, reaffirming PPRRRP’s mechanical synergy with natural shoulder kinematics. Its integration of orthogonally distributed passive prismatic joints (P_1_–P_3_) enables real-time accommodation of glenohumeral joint translations, converting interface constraints into compliant motions. In contrast, RRRUP’s reliance on a single universal joint introduces orientation-dependent stiffness, exacerbating force/torque fluctuations during tasks requiring rapid directional shifts. Statistical robustness was further evidenced by PPRRRP’s lower coefficient of variation (CV < 15%) across all metrics compared to RRRUP (CV > 35%), underscoring its reliability in clinical settings. The PPRRRP configuration’s design—prioritizing passive DOF allocation over active control—effectively decouples mechanical resistance from user intent, a critical factor in reducing compensatory movements and enhancing motor recovery efficacy. The clinical implications of these findings are profound: PPRRRP’s consistent force/torque profiles (Figure 13 and Figure 14) minimize user exertion, enabling longer, more effective rehabilitation sessions. RRRUP’s limitations, while not precluding basic functionality, highlight the risks of universal joints in multi-DOF exoskeletons, particularly for patients with limited proprioceptive feedback. Future iterations could integrate PPRRRP’s passive joint architecture with adaptive control algorithms to further optimize task-specific compatibility. These results collectively validate PPRRRP as a benchmark for rehabilitation exoskeletons, offering a template for balancing mechanical transparency with kinematic fidelity in human–robot interaction systems.

## 6. Discussion

This study systematically evaluated the kinematic performance of two shoulder rehabilitation exoskeleton configurations (PPRRRP and RRRUP) through dexterity, manipulability, and compatibility metrics, establishing PPRRRP as a superior design for high-precision rehabilitation tasks. The PPRRRP configuration demonstrated enhanced motion flexibility (***K***^−**1**^ > 0.7), near-isotropic manipulability (ellipsoid axis ratio < 1.5), and significantly lower interaction forces (${\bar{F}}_{total}=2.66\;N$) compared to RRRUP, validating its mechanical synergy with natural shoulder kinematics. This synergy reflects a biomimetic correspondence with the coordinated movement of the human shoulder complex, where translational and rotational motions are naturally coupled to maintain alignment and minimize internal stress. The PPRRRP design captures this biological mechanism through its passive adaptability and self-alignment capability. These findings advance the field by prioritizing passive joint integration over active control, a departure from conventional exoskeletons like ARMin-III [25] and Intelli-Arm [14], which rely heavily on complex actuation systems. Unlike prior studies that focused on active DOF optimization [12,15], this work demonstrates that strategic placement of passive prismatic joints can achieve kinematic compatibility without compromising user comfort or control simplicity—a critical advancement for clinical translation.

While the existing literature has extensively explored active compliance [9,10] and hybrid DOF architectures [16], few studies have quantified the relationship between passive joint allocation and task-specific performance. For instance, Jarrasse et al. [17] emphasized closed-chain analysis but overlooked transient joint dynamics, whereas Li et al. [18] proposed generalized DOF models without empirical validation. This study bridges these gaps by integrating real-time GH joint tracking (Figure 7) with statistically rigorous force/torque metrics (Figure 13 and Figure 14), offering a replicable framework for exoskeleton evaluation. This framework also embodies a biomimetic modeling concept, translating observed features of human biomechanics into quantitative design criteria. It links natural joint coordination with engineering evaluation, reinforcing the biological foundation of mechanical optimization.

The mechanical outcomes observed in this study align with established clinical and neurophysiological evidence. Reducing abnormal joint loading and preserving natural kinematic coupling have been shown to promote symmetrical muscle activation and enhance cortical plasticity during post-stroke rehabilitation. Such findings suggest that minimizing parasitic forces, as achieved by the PPRRRP configuration, may facilitate more efficient proprioceptive feedback and motor relearning. Therefore, the proposed exoskeleton not only replicates the mechanical features of the human shoulder but also supports neurophysiological principles essential for functional recovery.

However, several limitations warrant consideration. First, experiments were conducted on healthy participants, potentially underestimating performance variability in patients with motor impairments. Second, the evaluation focused on planar eating/combing tasks, neglecting three-dimensional activities like reaching behind the back. Third, long-term durability of passive joints under repetitive loading remains unverified. To address these, future studies should include clinical cohorts, expand task diversity, and conduct accelerated lifespan testing on mechanical components. A promising solution lies in merging PPRRRP’s passive architecture with adaptive control algorithms, enabling dynamic stiffness adjustment based on user capability. For example, integrating impedance control could further reduce interaction forces during abrupt motions. Additionally, extending the kinematic model to incorporate scapulothoracic joint dynamics may enhance trajectory prediction accuracy. New hypotheses emerge from these findings: (1) passive joint configurations may outperform active systems in energy efficiency for low-intensity rehabilitation; and (2) isotropic manipulability correlates with reduced muscle fatigue in prolonged use. Future research should test these through electromyography-assisted trials and metabolic cost analysis. Further progress may be achieved through functional biomimetic extensions that emulate muscle coordination and adaptive stiffness of the shoulder. Incorporating tendon-like elastic components or variable impedance mechanisms could enhance responsiveness and comfort in daily rehabilitation tasks.

In conclusion, this work redefines exoskeleton design paradigms by demonstrating that passive DOF optimization, rather than actuator proliferation, holds the key to naturalistic human–robot interaction. By addressing both mechanical and clinical constraints, the PPRRRP configuration sets a benchmark for next-generation rehabilitation robots, advocating for simplicity and adaptability in assistive technology.

## 7. Conclusions and Future Work

This study demonstrates that the PPRRRP exoskeleton configuration, through strategic integration of orthogonal passive prismatic joints, achieves superior kinematic compatibility by minimizing interaction forces (60% reduction vs. RRRUP) and maintaining near-isotropic manipulability (axis ratio < 1.5), outperforming conventional active/hybrid designs. Its mechanical transparency—prioritizing passive DOFs over actuator density—validates a paradigm shift in rehabilitation robotics, offering clinically viable low-torque operation (${\bar{T}}_{total}=0.18\;N\cdot m$) and enhanced user comfort. Methodologically, the fusion of Hunt’s formula with task-specific Jacobian analysis bridges theoretical synthesis and empirical validation, providing a replicable framework for compatibility assessment. Future efforts should prioritize clinical validation with motor-impaired cohorts, hybrid control systems integrating adaptive impedance modulation, and expanded kinematic models incorporating scapulothoracic dynamics for 3D task optimization. Hypotheses on energy efficiency advantages of passive architectures and isotropy–fatigue correlations warrant investigation via electromyography and metabolic cost analysis. By redefining design priorities toward mechanical synergy over actuation complexity, this work establishes foundational principles for next-generation exoskeletons that harmonize with—rather than constrain—human biomechanics, advocating for simplicity in assistive technology development.

## Figures and Tables

**Table 1:** Potential mechanism configurations of the shoulder rehabilitation.

Amount of Active Joints	Amount of Passive Joints	Configurations of Sub-Chains
3	3	3R_a_3R, 3R_a_3P, 3R_a_2P1R, 3R_a_2R1P
3	2	3R_a_1P1U, 3R_a_1R1U, 3R_a_1P1C, 3R_a_1R1C
3	1	3R_a_1S

**Table 2:** The technical specifications of the sensors and data acquisition hardware.

Torque Sensor		Data Acquisition	
Specification	Details	Specification	Details
Model	Mini45-SI-145-5	Model	ATI Net F/T DAQ System
Measurement Range	F_x_,F_y_ (± 145N); F_z_ (±290 N); T_x_,T_y_,T_z_ (±5 Nm)	Sampling Rate	7000 samples/second per channel
Uncertainty	±0.5% of full scale	Uncertainty	±0.01% of reading
Bandwidth	1000 Hz	Resolution	16-bit
Resolution	0.01 N; 0.001 Nm	Input Range	±10 V
Nonlinearity	<±0.2% of full scale	Communication Interface	Ethernet, USB, Serial

**Table 3:** Parameters associated with the human–robot closed chain.

Name	*l*_1_	*l*_2_	*l*_3_	*l*_4_	*l*_5_	*l*_6_	*l*_7_	*l*_8_	*l*_9_	*l*_10_	*l*_11_
Numeric (mm)	40	100	100	400	300	260	400	400	280	120	254
Name	*l*_12_	*l*_13_	*l*_14_	*l*_15_	*l*_16_	*l*_17_	*l*_18_	*l*_19_	*x*_0_	*y*_0_	*z*_0_
Numeric (mm)	400	300	260	400	400	160	80	254	156	136	36

## Data Availability

The datasets generated and analyzed during the current study are available from the corresponding author upon reasonable request. The processed data and analysis scripts supporting the findings of this study will be deposited in an open-access institutional repository following the paper’s acceptance to ensure transparency and future reuse.

## Appendix A

1. PPRRRP configuration

$$\begin{array}{c} T_{1,m}^{1,s}=Trans(X_{1,s},a_{0})\cdot Trans(Y_{1,s},b_{0})\cdot Trans(Z_{1,s},c_{0})\cdot Rot\left(Z_{1,s},-90^{{}^{\circ}}\right)\cdot Rot\left(X_{1,s},90^{{}^{\circ}}\right)\\ T_{1,0}^{1,m}=Trans\left(Z_{1,m},-l_{1}\right)\cdot Trans\left(Z_{1,0},s_{1,1}\right)\\ T_{1,1}^{1,0}=Trans\left(Z_{1,0},-l_{2}\right)\cdot Trans\left(Z_{1,1},s_{1,2}\right)\\ T_{1,2}^{1,1}=Trans\left(Z_{1,1},-l_{3}\right)\cdot Rot\left(Y_{1,1},-90^{{}^{\circ}}\right)\cdot Rot\left(Z_{1,1},90^{{}^{\circ}}\right)\cdot Rot\left(Z_{1,2},\theta_{1,1}\right)\\ T_{1,3}^{1,2}=Trans\left(Y_{1,2},l_{4}\right)\cdot Trans\left(Z_{1,2},-l_{5}\right)\cdot Rot\left(Z_{1,2},-90^{{}^{\circ}}\right)\cdot Rot\left(Y_{1,2},90^{{}^{\circ}}\right)\cdot Rot\left(Z_{1,3},\theta_{1,2}\right)\\ T_{1,4}^{1,3}=Trans\left(Y_{1,3},l_{6}\right)\cdot Trans\left(Z_{1,3},-l_{7}\right)\cdot Rot\left(Z_{1,3},90^{{}^{\circ}}\right)\cdot Rot\left(Y_{1,3},-90^{{}^{\circ}}\right)\cdot Rot\left(Z_{1,4},\theta_{1,3}\right)\\ T_{1,5}^{1,4}=Trans\left(X_{1,4},-l_{8}\right)\cdot Trans\left(Z_{1,4},-l_{9}\right)\cdot Rot\left(Z_{1,4},-90^{{}^{\circ}}\right)\\ T_{1,g}^{1,c}=Rot\left(Z_{1,c},\alpha \right)\cdot Rot\left(Y_{1,c},\beta \right)\cdot Rot\left(Z_{1,c},\gamma \right)\\ T_{1,6}^{1,g}=Trans\left(X_{1,g},-l_{11}\right)\cdot Trans\left(Z_{1,g},l_{10}\right) \end{array}$$

where α,β, and γ denote the three Euler angles of upper limb motion from the ISB definition. The $s_{1,1}$ and $s_{1,2}$ denote the linear displacement of joint P_1_ and P_2_.

2. RRRUP configuration

$$\begin{array}{c} T_{2,0}^{2,s}=Trans(X_{2,s},a_{1}\cdot Trans(Y_{2,s},b_{1})\cdot Trans(Z_{2,s},c_{1})\cdot Rot\left(Z_{2,s},-90^{{}^{\circ}}\right)\cdot Rot\left(X_{2,s},90^{{}^{\circ}}\right)\\ T_{2,1}^{2,0}=Trans\left(X_{2,0},-l_{13}\right)\cdot Rot\left(X_{2,1},\theta_{2,1}\right)\\ T_{2,2}^{2,1}=Trans\left(Z_{2,1},l_{12}\right)\cdot Trans\left(X_{2,1},-l_{13}\right)\cdot Rot\left(Z_{2,1},90^{{}^{\circ}}\right)\cdot Rot\left(Y_{2,1},-90^{{}^{\circ}}\right)\cdot Rot\left(Y_{2,2},\theta_{2,2}\right)\\ T_{2,3}^{2,2}=Trans\left(Z_{2,2},-l_{14}\right)\cdot Trans\left(Y_{2,2},-l_{15}\right)\cdot Rot\left(Z_{2,2},90^{{}^{\circ}}\right)\cdot Rot\left(Y_{2,2},-90^{{}^{\circ}}\right)\cdot Rot\left(Y_{2,3},\theta_{2,3}\right)\\ T_{2,4}^{2,3}=Trans\left(Z_{2,3},-l_{16}\right)\cdot Trans\left(Z_{2,4},s_{2,1}\right)\\ T_{2,5}^{2,4}=Trans\left(X_{2,4},l_{17}\right)\\ T_{2,g}^{2,c}=Rot\left(Z_{2,c},\alpha \right)\cdot Rot\left(Y_{2,c},\beta \right)\cdot Rot\left(Z_{2,c},\gamma \right)\\ T_{2,6}^{2,g}=Trans\left(X_{2,g},-l_{19}\right)\cdot Trans\left(Z_{2,g},l_{18}\right) \end{array}$$

where α,β, and γ denote the three Euler angles of upper limb motion from the ISB definition. The $s_{2,1}$ denote the linear displacement of joint P_2_.
